# Dietary Patterns in New Zealand Women: Evaluating Differences in Body Composition and Metabolic Biomarkers

**DOI:** 10.3390/nu11071643

**Published:** 2019-07-18

**Authors:** Shakeela N. Jayasinghe, Bernhard H. Breier, Sarah A. McNaughton, Aaron P. Russell, Paul A. Della Gatta, Shaun Mason, Welma Stonehouse, Daniel C.I. Walsh, Rozanne Kruger

**Affiliations:** 1 School of Sport, Exercise and Nutrition, College of Health, Massey University, Auckland 0745, New Zealand; 2 Riddet Institute, Centre of Research Excellence, Palmerston North 4442, New Zealand; 3 Institute for Physical Activity and Nutrition, School of Exercise and Nutrition Sciences, Deakin University, Melbourne 3125, Australia; 4 Health and Biosecurity, Commonwealth Scientific and Industrial Research Organization, Adelaide, South Australia 5000, Australia; 5 School of Natural and Computational Sciences, Massey University, Auckland 0745, New Zealand

**Keywords:** dietary patterns, refined and processed foods, metabolic biomarkers, body composition, macronutrient intake, premenopausal women

## Abstract

The combinations of food consumed together (dietary patterns) may have a greater influence on health than nutrients or food groups consumed independently. This study investigated the relationship between dietary patterns, body composition and metabolic biomarkers of premenopausal New Zealand women from three ethnic groups. In total, 408 New Zealand European, Māori and Pacific women aged 16–45 years participated in the Women’s EXPLORE (EXamining Predictors Linking Obesity Related Elements) study. Participants completed a 220-item food frequency questionnaire. Several body composition parameters and metabolic biomarkers were measured. Dietary patterns were extracted by principal component analysis and dietary pattern scores were categorised into tertiles to assess links with other measured parameters. Women with higher scores for the ‘refined and processed’ pattern were younger, had higher body mass index, total body fat, plasma leptin and plasma insulin (*p* < 0.001), and lower plasma ghrelin levels (*p* < 0.05) than women with lower scores. In addition, more Māori (51%) and Pacific (68%) women followed the ‘refined and processed’ pattern, while more New Zealand European women (40%) followed the ‘sweet and savoury snacking’ pattern. These data show that dietary pattern analysis is a useful tool to assess links between diet and metabolic health. It further reveals interesting ethnic group-specific differences in dietary pattern use.

## 1. Introduction

Dietary intake is inherently complex; the cumulative effects of various nutrients gained from eating different foods can be either beneficial or harmful to health [1]. Dietary pattern analysis has emerged as a complementary method of characterising dietary intakes [2]. Dietary patterns represent a broader picture of the overall diet as they reflect the combinations of foods and associated nutrients consumed together [2,3]. Principal component analysis (PCA), a well-established method of factor analysis derives dietary patterns based on the inter-correlations between food groups that are frequently consumed together. With PCA, each individual is given a score reflective of their intake for a dietary pattern, and the dietary pattern score can be used to differentiate between individuals with high versus low intakes. Furthermore, there is also the advantage of assessing differences in measured parameters (e.g., body composition, metabolic biomarkers) between individuals with different intakes for a dietary pattern [4].

Dietary pattern analysis is widely used in studies to assess the link between diet and nutrition-related health outcomes [5,6]. In general, higher intakes of a ‘Western’ or ‘unhealthy’ dietary pattern characterised by refined grains, take-away food, red and processed meats and sugar-sweetened beverages have been associated with higher body weight, body mass index (BMI) and adiposity [5,7]. On the other hand, following a ‘Prudent’ or ‘healthy’ dietary pattern characterised by fruits, vegetables, wholegrains, eggs, fish and seafood has been associated with a lower BMI and adiposity, a smaller increase in BMI and waist circumference, and lower inflammation [5,6,7].

Limited studies have explored dietary patterns in New Zealand (NZ) populations [8,9,10]. Two of these studies were conducted in pregnant women, and the findings cannot be generalised to other population groups [9,10]. Another study using 24-h diet recall data from a sample of 4657 adults reported that a ‘healthy’ dietary pattern was positively associated with age, female gender and New Zealand European ethnic group and negatively associated with BMI and waist circumference [8]. Furthermore, a ‘traditional’ dietary pattern was positively associated with male gender, smoking and food insecurity [8]. However, there is a dearth of NZ studies that have explored the links between specific dietary patterns, obesity and metabolic health biomarkers, in particular the link with endocrine regulators of adiposity, appetite and inflammation. 

Furthermore, obesity rates in NZ have increased substantially over the past three decades, with some population groups having a higher prevalence of obesity (Māori and Pacific women 50% and 69% obese, respectively) compared to the overall population (34% obese) [11]. Although the aetiology of obesity is complex, it is important to identify specific dietary determinants that may increase the risk of obesity. According to the NZ Adult Nutrition Survey, there were no differences in intake of total energy and energy from protein or carbohydrate between Māori and non-Māori women [12]. However, Māori women consumed a higher mean percentage of energy from total fat, saturated fat and monounsaturated fat than non-Māori women [12]. Pacific women had a higher total energy intake and higher energy intake from monounsaturated fats than non-Pacific women [13], but there were no differences in energy intake from protein or carbohydrate between Pacific and non-Pacific women. However, it has been reported that there are differences in dietary habits and cooking practices between ethnic groups in NZ [12,13]. For example, Māori and Pacific women were more likely to eat fast-food, were more likely to consume soft drinks and energy drinks, and were less likely to eat breakfast daily, choose reduced fat or trim milk, trim fat off meat and meet guidelines for vegetable and fruit intake compared to non-Māori and non-Pacific women, respectively [11,12,13]. Therefore, understanding differences in specific dietary parameters between ethnic groups may contribute towards understanding obesity and metabolic disease risks.

The aim of the present study was to investigate the relationship between dietary patterns, body composition and metabolic health biomarkers in a cohort of premenopausal NZ women from three ethnic groups (New Zealand European (NZE), Māori and Pacific). This study builds on our previously published work, which explored the associations between dietary patterns and body composition of only the NZE cohort of the women’s EXPLORE (EXamining Predictors Linking Obesity Related Elements) study [14]. In the present study, we generated dietary patterns for the overall study population (including all three ethnic groups) and assessed the links with body composition, metabolic biomarkers and endocrine regulators of adiposity, appetite and inflammation. A further analysis was done within each dietary pattern to investigate the association between the ethnic groups and dietary patterns.

## 2. Materials and Methods

### 2.1. Study Population

We used data from the cross-sectional women’s EXPLORE study, designed to investigate the metabolic disease risks associated with different body fat profiles of NZ women from three ethnic groups [15]. Premenopausal women from NZE (*n* = 233), Māori (*n* = 84) and Pacific (*n* = 91) ethnic groups aged 16–45 years participated in the study. Women were excluded if they were pregnant or breastfeeding, had irregular menstrual cycles, had been diagnosed with any chronic illness or were taking medication that may influence taste perception. Recruitment from the urban Auckland area was conducted using various methods, including advertising in newspapers, magazines, social media, email lists and through community groups. The EXPLORE study was conducted in accordance with the Declaration of Helsinki, and the study protocol was approved by the Massey University Human Ethics Committee (Southern A, Application 13/13). This study was registered in the Australian New Zealand Clinical Trials Registry as ACTRN12613000714785. All participants provided informed written consent prior to participation.

### 2.2. Study Procedure

The study had two phases; phase 1 (screening) and phase 2 (data collection). A screening questionnaire was used to determine the participant’s initial eligibility of age, ethnic group and health. Participants who fit the initial eligibility were screened at Massey University in Auckland or at an off-site location for height (stadiometer), weight and body fat (Bioelectrical Impedance Analysis, Inbody 230, Biospace Co. Ltd, Seoul, Korea), to determine their eligibility as required by the EXPLORE study design [15]. Phase 2 data collection was conducted at the Human Nutrition Research Unit at Massey University in Auckland. Phase 2 visits were scheduled during the first 14 days of the menstrual cycle to standardise effects of menstrual cycle hormones on food intake [16]. To standardise hunger and diurnal hormonal fluctuations [17], phase 2 visits were conducted between 7 a.m. and 9.30 a.m. following an overnight fast. Body composition measurements, fasting blood samples and dietary intake data for each individual were obtained on the same day.

### 2.3. Assessment of Body Composition

All anthropometric measurements were obtained using the International Society for the Advancement of Kinanthropometry (ISAK) protocols [18]. Body weight was measured to the nearest 0.1 kg using electronic scales, and height was measured with a Harpenden stadiometer to the nearest 0.1 cm. Waist circumference (WC) and hip circumference (HC) were measured according to the ISAK protocol, and waist to hip circumference ratios (WHRs) were calculated [18]. Total body fat was measured by full body composition analysis using an air displacement plethysmography device (BodPod, 2007A, V4.2+ software, Life Measurement Inc., Concord, CA, USA) [19]. Android and gynoid fat were determined from whole body dual-energy X-ray absorptiometry (Hologic QDR Discovery A, Hologic Inc., Bedford, MA, USA).

### 2.4. Assessment of Metabolic and Endocrine Biomarkers

Fasting blood samples were drawn into ethylenediaminetetraacetic acid (EDTA) and serum vacutainer tubes by qualified phlebotomists. An aliquot of EDTA whole blood was immediately frozen at −80 °C for glycosylated haemoglobin (HbA1c) analysis. Within an hour of collection, vacutainer tubes were centrifuged at 3500 rpm at 4 °C for 15 min, and aliquots were frozen at −80 °C. Serum insulin was measured by the ADVIA Centaur system, using a chemiluminescent two-site sandwich immunoassay method (Siemens Healthcare Diagnostics Inc., Tarrytown, NY, USA, catalogue # 02230141-128434) [20]. Using standard automated laboratory procedures of the Dimension Vista system (Siemens Healthcare Diagnostics Inc., Tarrytown, NY, USA), levels of serum total cholesterol (catalogue # K1027) [21], C-reactive protein (CRP), high-density lipoprotein cholesterol (HDL-C, catalogue # K3048) [22], glucose (catalogue # K1039) [23] and triglyceride (catalogue # K2069) [21] were measured. Serum low-density lipoprotein cholesterol (LDL-C) levels were calculated using measured variables. The HbA1c levels were measured using the high-performance liquid chromatography method (Bio-Rad D100, Bio-Rad Laboratories, Hercules, CA, USA) [24].

Milliplex immunoassay kits (Millipore Corp., Billerica, MA, USA) were used to simultaneously measure plasma levels of interleukin-6 (IL-6), interleukin-10 (IL-10) and tumour necrosis factor-alpha (TNF-α) (catalogue # HSTCMAG-28SK), and ghrelin and leptin (catalogue # HMHEMAG-34K) as previously described [25]. Mean inter-assay coefficients of variation for the 5 factors analysed were as follows: IL-6—5.5%; IL-10—5.1%; TNF-α—4.7%; ghrelin—5.7%; leptin—3.7%. Intra-assay coefficient of variation was determined by replicate analysis (n = 11) of the provided assay quality controls, and the results were as follows: IL-6—7.8%; IL-10—7.7%; TNF-α—6.9%; ghrelin—5.9%; leptin—4.7%.

### 2.5. Dietary Assessment

A validated 220-item semi-quantitative food frequency questionnaire (FFQ) was used to assess the frequency of foods and beverages consumed over the previous month [26]. The FFQ contained food items organised by common food groups such as breads and cereals, meat, fish, poultry, fats and oils, fruits and vegetables and take-away foods. Standard natural portions (e.g., 1 medium banana) or standard volumes (e.g., 1 pottle of yoghurt) were used to measure the intakes of the food items. Participants were asked to select the intake frequency of the standard serving size for each food item that best described their usual intake over the previous month, from nine frequency categories (never to more than four times per day). The food items in the FFQ were adapted from the 2007/08 NZ Adult Nutrition Survey and included culturally specific foods of Māori and Pacific ethnic groups (e.g., Rēwena bread, pipi) [27]. All data obtained from the FFQ were entered into FoodWorks 7 (FoodWorks professional 2013, Xyris Software, Queensland, Australia) using the NZ food composition database to determine energy and macronutrient intakes.

### 2.6. Dietary Pattern Extraction

The frequencies of intake of the individual food items of the FFQ were firstly converted to daily frequency equivalents (DFEs), calculated by allocating proportional values to the original frequency categories with reference to a base value of 1.0, equivalent to once a day [28]. The scores were calculated as: DFE score of 0—never; 0.008—less than once per month; 0.067—1–3 times per month; 0.14—once per week; 0.36—2–3 times per week; 0.71—4–6 times per week; 1—once a day; 2.5—2–3 times per day; 4—more than four times per day. Based on the nutritional composition, food items were categorised into 57 food groups (e.g., sweetened milk products, starchy vegetables, red meats) (Supplementary Table S1). Individual items that solely created a food group (e.g., potatoes, yoghurt) were consumed by at least 10% of the study participants. For each participant, a single DFE score for each of the 57 food groups was calculated.

Dietary patterns in the present study were determined using the methodology previously published for NZE women of the EXPLORE study [14]. However, in the present study, dietary patterns were re-extracted using data from the overall population including all three ethnic groups. Briefly, dietary patterns were extracted by the PCA method and orthogonal (varimax) rotation using the participant’s DFE scores for each of the 57 food groups. The number of dietary patterns that best suited the data were determined using scree plots, an eigenvalue cut-off >1 and the factor loadings. The extracted dietary patterns were named based on the characteristics of the food groups with the highest positive factor loadings (>0.3), indicating a greater contribution to the pattern. A negative loading (>−0.3) indicated a strong inverse relationship between the food group and dietary pattern. The Kaiser–Meyer–Olkin measure of sampling adequacy was 0.7 (>0.5 acceptable), and Bartlett’s Test *p* value was <0.001 (<0.001 acceptable) [29]. Inter-item reliability of each dietary pattern was assessed using Cronbach’s α (≥0.7 = good, 0.5–0.7 = moderate) [29] to ensure that the associated food groups were an appropriate measure of the dietary pattern. A further check was done to assess whether the inter-item reliability of the dietary pattern would increase if any food group was removed. In our study, the inter-item reliability of the dietary patterns did not improve with the removal of any food group.

### 2.7. Data Handling and Statistical Analysis

Four participants were excluded due to incomplete FFQ data, and a further 10 and 14 participants were excluded due to under-reporting (<1000 kcal/day) and over-reporting (>5000 kcal/day), respectively [30,31]. Following blood analysis, two participants were excluded due to their HbA1c levels being higher than 50 mmol/mol [32]. A final sample size of 378 was used for data analysis.

All data were analysed using SPSS software version 24 (IBM corp., Armonk, NY, USA). Continuous variables were tested for normality, and non-normal data were log transformed and re-tested for normality. Log transformed data were used for leptin, CRP, IL-6 and IL-10. Differences in measured parameters between the ethnic groups were tested using one-way analysis of covariance (with the Bonferroni correction). Dietary pattern scores were categorised into tertiles to differentiate women with lower scores (i.e., tertile 1, lower intake) from higher scores (i.e., tertile 3, higher intake). The differences in body composition, macronutrient intake and metabolic biomarkers between the dietary pattern tertiles were tested using analysis of covariance and post hoc tests (with the Bonferroni correction) to identify where the differences lay. The covariates used in the analysis include ethnic group, age and energy intake, as these factors are known to influence diet and body composition [9,33]. The relationship between categorical variables (i.e., ethnic group and dietary pattern tertiles) were assessed using chi-square test together with standardised residuals to test significant differences between the groups. The level of significance was assessed using the standardised residual values by the criteria; ±1.96 = *p* < 0.05; ±2.58 = *p* < 0.01; ±3.29 = *p* < 0.001 [29]. Continuous variables are reported as mean ± standard error of the mean (SEM), and categorical data are reported as *n* (%). All tests were two-tailed, and a *p* < 0.05 was considered statistically significant.

## 3. Results

### 3.1. Characteristics of Study Participants

Characteristics of study participants are reported in Table 1. Overall, the participants were young (31 years), and the majority belonged to the NZE ethnic group (59%). In terms of BMI groups, the majority of NZE women were of normal-weight BMI, and the majority of Pacific women were in the obese BMI group. In terms of body fat, the majority of NZE women had <35% body fat, while the majority of Māori (51%) and Pacific (63%) women had >35% body fat.

### 3.2. Dietary Patterns

In the present study, using the PCA method and varimax rotation, four distinct dietary patterns were identified for the overall population (including all three ethnic groups). The factor loadings of the food groups contributing to the four dietary patterns are reported in Table 2. Dietary pattern one, named ‘refined and processed’, explained 9% of the variance in food intake. This pattern was characterised by high factor loadings on crumbed and deep-fried food, fast-food, puddings, fruit drinks, soft drinks and other beverages, sweetened milk products, refined grains, starchy vegetables, white breads and sweetened cereals. The ‘refined and processed’ pattern was also characterised by negative loadings for water and nuts and seeds. Pattern two, labelled ‘sweet and savoury snacking’, explained 7% of the variance in food intake and was characterised by high loadings on cakes and biscuits, sweet and savoury spreads, sweet and savoury snacks, margarine, peanut butter and peanuts, sauces, whole grain breads, cheese and crackers. Pattern three explained 5% of the variance in food intake and was named ‘fruit and vegetable’. This pattern had high factor loadings on all fruits and vegetables (except potatoes), soy products, legumes, wholegrains and yoghurt. Pattern four, ‘fats and meat’, explained 4% of the variance in food intake and was characterised by high loadings on fats, all red, white and processed meats, creamy dressings, egg dishes, coconut fats, alcoholic beverages and full-fat milk, and negative loadings on low-fat milk (Table 2). The inter-item reliability of the dietary patterns indicated good reliability for patterns one, two and three (Cronbach’s α = 0.7) and moderate reliability for pattern four (Cronbach’s α = 0.5).

### 3.3. Participant Characteristics of Dietary Pattern Tertiles

Women with higher scores for the ‘refined and processed’ pattern were younger, had higher body weight, BMI, total body fat %, android fat %, WC, HC and WHR than women with lower scores, even after controlling for energy intake (all *p* < 0.001, Table 3). Furthermore, there were significantly more Māori (51%, *p* < 0.05) and Pacific (68%, *p* < 0.001) women and significantly fewer NZE women (16%, *p* < 0.001) in tertile 3 compared to tertile 1 of the ‘refined and processed’ pattern. Women with higher scores for the ‘sweet and savoury snacking’ pattern had higher body weight, total body fat %, WC and HC than women with lower scores (all *p* < 0.05). However, these differences were no longer significant after controlling for energy intake. There were significantly more NZE women (40%, *p* < 0.01) and significantly fewer Māori women (15%, *p* < 0.001) in tertile 3 compared to tertile 1 of the ‘sweet and savoury snacking’ pattern.

There were no significant differences in age, ethnic group or body composition measurements between tertiles of the ‘fruit and vegetable’ pattern, even after controlling for energy intake. Women with higher scores for the ‘fats and meat’ pattern had higher body weight, BMI, android fat %, WC and HC compared to women with lower scores (*p* < 0.005). However, these differences were no longer evident after controlling for energy intake. Furthermore, there were significantly fewer NZE women (27%, *p* < 0.01) and significantly more Māori women (47%, *p* < 0.01) in tertile 3 compared to tertile 1 of the ‘fats and meat’ pattern (Table 3).

### 3.4. Energy and Macronutrient Intakes of Dietary Pattern Tertiles

All dietary pattern tertiles were within the acceptable macronutrient distribution range (AMDR) for protein intake (15–25%, Table 4) [36]. Interestingly, all dietary pattern tertiles were closer to or slightly above the upper limit of the AMDR for fat intake (20–35%), and were closer to or slightly lower than the lower limit of the AMDR for carbohydrate intake (45–65%) [36].

Women with higher scores for the ‘refined and processed’ pattern had significantly higher intakes of total energy (*p* < 0.001), percentage carbohydrate (*p* < 0.001), starch (*p* < 0.002), total sugar (*p* < 0.05) and sucrose (*p* = 0.002) and significantly lower intakes of percentage fat and protein (*p* < 0.004) compared to women with lower scores (Table 4). Women with higher scores for the ‘sweet and savoury snacking’ pattern had significantly higher intakes of total energy and percentage starch (*p* < 0.001) and significantly lower intakes of percentage protein (*p* < 0.001), glucose (*p* = 0.001) and fructose (*p* = 0.01) than women with lower scores (Table 4).

With regards to the ‘fruits and vegetable’ pattern, women with higher scores had significantly higher intakes of total energy (*p* < 0.001), percentage protein (*p* < 0.05), carbohydrate (*p* < 0.05), total sugar (*p* < 0.001), glucose (*p* < 0.001) and fructose (*p* < 0.001) and a significantly lower intake of percentage fat (*p* < 0.001) compared to women with lower scores (Table 4). Women with higher scores for the ‘fats and meat’ pattern had significantly higher intakes of total energy (*p* < 0.001), percentage protein and fat (*p* < 0.001) and significantly lower intakes of percentage carbohydrate, starch, total sugar and fructose (all *p* < 0.005) than women with lower scores (Table 4).

### 3.5. Metabolic and Endocrine Biomarkers of Dietary Pattern Tertiles

Women with higher scores (tertile 3) for the ‘refined and processed’ pattern had significantly higher levels of insulin and leptin (*p* < 0.001) and lower levels of ghrelin (*p* = 0.03) and HDL-C (*p* < 0.001) compared to women with lower scores (tertile 1) (Table 5). Furthermore, women with higher scores for the ‘sweet and savoury snacking’ pattern had a significantly higher level of leptin (*p* = 0.007) compared to women with lower scores. We observed no significant differences in metabolic biomarkers between tertiles of the ‘fruit and vegetable’ and ‘fats and meat’ patterns (Table 5).

## 4. Discussion

In the present study, we identified four dietary patterns explaining 25% of the variance in food intake; refined and processed, sweet and savoury snacking, fruit and vegetable, and fats and meat. The total percentage variance explained by the four dietary patterns of our study (25%) was similar to a study done in pregnant NZ women that identified four dietary patterns (junk, health conscious, traditional/white bread, fusion/protein) explaining 23.4% variance [9], but higher than a recent study in men and women that identified two dietary patterns (healthy, traditional) explaining 12% of the variance in food intake [8]. The percent variance explained by individual dietary patterns is a function of the number of variables included in the factor analysis (i.e., food groups) and the correlation matrix [37,38]. Therefore, we would expect differences in variances explained by the different dietary patterns between studies.

The dietary patterns found in the present study were similar to the patterns found in our previous publication (i.e., healthy, snacking, fruit and vegetable, energy-dense meat patterns) [14], with the exception of the very distinct ‘refined and processed’ dietary pattern found in the present study, which overshadowed the ‘healthy’ dietary pattern found previously. Furthermore, we also found that the factor loadings between the food groups and the dietary patterns were stronger in the present study compared to our previous publication. We believe the differences in dietary patterns between the two studies may be attributed to the more varied diet introduced by the larger sample size (i.e., inclusion of women from all three ethnic groups) of the present study.

The aim of the present study was to investigate the relationship between dietary patterns, body composition and metabolic biomarkers in a cohort of premenopausal NZ women from three ethnic groups. There are two main findings in our study. Firstly, women with higher scores for the ‘refined and processed’ dietary pattern had higher BMI and adiposity, higher circulating levels of insulin and leptin, and lower levels of ghrelin compared to those with lower scores. Secondly, we found ethnic group-specific differences in dietary patterns, where more Māori and Pacific women followed the ‘refined and processed’ pattern, while more NZE women followed the ‘sweet and savoury snacking’ pattern.

### 4.1. The Link between the ‘Refined and Processed’ Dietary Pattern and Obesity

The ‘refined and processed’ pattern was characterised by foods that are refined, highly processed and energy-dense. The ‘refined and processed’ dietary pattern of our study explained 9% of the variance in food intake and was similar to dietary patterns of other studies such as the ‘Western’ pattern, which explained 10% of the variance [5], and the ‘fast-food/dessert’ [39] and ‘refined grains and dessert’ patterns [40], which explained 13% of the variance in food intake. Consistent with others, women with higher scores for the ‘refined and processed’ pattern in our study had higher intakes of total energy, percentage carbohydrate, starch, total sugar and sucrose, and a lower intake of percentage protein compared to women with lower scores [39,41].

We also found that women with higher scores for the ‘refined and processed’ pattern were younger and had higher body weight, BMI, total body fat %, android fat %, WC, HC and WHR than women with lower scores, even after controlling for energy intake. Furthermore, women with higher scores for the ‘refined and processed’ pattern had higher circulating concentrations of insulin and leptin and lower plasma levels of ghrelin and HDL-C compared to women with lower scores. It has been proposed that refined carbohydrates and sugars promote hyper-insulinaemia, which drives glucose and fatty acids into adipose tissue, resulting in preferential deposit of ingested calories as fat [42]. Our data suggest that higher energy and carbohydrate (starch and sugar) intakes of the ‘refined and processed’ pattern may drive hyper-insulin secretion and increased adiposity, leading to hyper-leptinaemia, as reported in previous studies describing the pathogenesis of obesity [43,44]. In the present study, it is important to note that the increase in insulin secretion appears to be sufficient to achieve normal plasma glucose and HbA1c concentrations [32].

In our study, it is possible that the lower plasma ghrelin of women with higher scores for the ‘refined and processed’ pattern may represent a physiological adaptation to reduce appetite to the high intake of refined carbohydrates and sugar. However, the reduction in plasma ghrelin may not be sufficient to reduce appetite in the face of hyper-leptinaemia [45]. Furthermore, lower HDL-C levels in women with higher scores for the ‘refined and processed’ pattern may represent an early indication of disturbed lipid metabolism [46]. However, other lipid and inflammatory markers were not different between the ‘refined and processed’ pattern tertiles, which may be attributed to the study participants being generally healthy and of premenopausal age.

### 4.2. Ethnic Group-Specific Differences within Dietary Patterns

Interestingly, we found ethnic group-specific differences within dietary patterns. One of the important findings was that more Māori (51%) and Pacific (68%) women and fewer NZE (16%) women followed the ‘refined and processed’ pattern. In line with the findings of our study, the 2008/09 NZ Adult Nutrition Survey reported that Māori and Pacific women were three times more likely to eat fast-food and were 1.5 times more likely to consume soft drinks and energy drinks three or more times a week compared to non-Māori and non-Pacific women, respectively [12,13]. In our study, we observed higher levels of adiposity and plasma insulin levels in Māori and Pacific women (data presented in Supplementary Table S2). Furthermore, although within the normal healthy range, Pacific women had higher glucose and HbA1c levels than NZE women (data presented in Supplementary Table S2), indicating an early sign of impaired glucose metabolism [32]. We could speculate that the endocrine and metabolic consequences of consuming dietary patterns such as the ‘refined and processed’ pattern may be a significant dietary contributor to higher metabolic disease and obesity risk profiles observed in Māori and Pacific ethnic groups [11]. Moreover, socio-economic inequities and food insecurity have been clearly documented for Māori and Pacific ethnic groups in comparison to other NZ population groups [47,48,49]. The finding that more Māori and Pacific women were following the ‘refined and processed’ dietary pattern which characterises foods that are easily accessible and more affordable but energy-dense and often nutrient-poor (e.g., fast-food, soft drinks) [12,13], may explain, at least in part, the differences in obesity and metabolic disease risk influenced by socio-economic aspects [50,51].

We also found that more NZE women (40%) followed the ‘sweet and savoury snacking’ pattern. In our study, women with higher scores for the ‘sweet and savoury snacking’ pattern had higher intakes of total energy and percentage starch, and lower intakes of percentage glucose, fructose and protein than women with lower scores. It has been discussed that the increasing prevalence of snacking is considered an important influence on health, as the types of foods and beverages consumed as snacks often contribute towards higher intakes of energy and added sugars [52]. Therefore, more research is needed to further understand the relationship between snacking habits, diet quality and metabolic health in different population groups in NZ.

### 4.3. The Link between Other Dietary Patterns and Metabolic Health

In our study, women with higher scores for the ‘sweet and savoury snacking’ and ‘fats and meat’ patterns had higher body composition measurements (e.g., BMI, total body fat) than women with lower scores. However, these differences were no longer significant after adjusting for energy intake. In addition, metabolic biomarkers were not different between the dietary patterns tertiles. The lack of body composition and metabolic biomarker differences could be attributed to the lower variance in food intake explained by these dietary patterns, as it has been discussed that dietary patterns explaining larger variances in food intake more strongly relate to health outcomes [53].

Women with higher scores for the ‘fruit and vegetable’ pattern had higher intakes of percentage protein, carbohydrate, total sugar, glucose and fructose and a lower intake of percentage fat compared to those with lower scores, indicating a generally healthy dietary composition. We found no differences in age, body composition or metabolic biomarkers between the ‘fruit and vegetable’ pattern tertiles. These findings contrast with studies that show links between higher intakes of similar dietary patterns (e.g., ‘fruit, vegetables and dairy’, ‘prudent’) and lower BMI, body weight, fat mass, WC, HDL-C levels and insulin levels [5,6,8,54,55]. The differences in findings between the studies could be attributed to the additional food groups (e.g., dairy, fish, poultry) present in dietary patterns of other studies, which constitute food groups of a more balanced diet [5,55]. In addition, although energy and total sugar were higher in women with higher scores for the ‘fruit and vegetable’ pattern in our study, total sugar mostly consisted of natural sugars from fruit and vegetables rather than added sugars (i.e., sucrose), representing the healthier type of sugars.

### 4.4. Strengths, Limitations and Future Directions

The present study has several strengths. Dietary patterns in our study were derived using a well-established data-driven statistical method, and the dietary patterns identified were similar to patterns found in other studies [5,6]. Furthermore, the total percentage variance explained by the four dietary patterns of our study (25%) was higher than the variance explained by other studies done in NZ [8,9,10]. We also used several measures of body composition (BMI, total and regional body fat) and a range of metabolic and endocrine biomarkers of adiposity, appetite and inflammation to assess the link between dietary patterns and metabolic health. Previous NZ studies have only investigated associations between dietary patterns, socio-demographic factors, lifestyle factors and a limited number of anthropometric measurements (e.g., BMI, waist circumference) [8,9,10,14]. In addition, we assessed ethnic group-specific differences within each dietary pattern to identify specific dietary parameters that increase the risk of obesity and metabolic disease [11].

Several limitations of the present study warrant further investigation. Firstly, there was disproportionate sampling from each ethnic group due to the challenges encountered during recruitment. Therefore, we were not able to investigate the relationship between dietary patterns and metabolic health markers within each ethnic group separately. However, we observed clear ethnic group-based differences within dietary patterns that require further investigation. Secondly, the study participants were a convenience sample of women living in Auckland, which limits the generalisability of the findings. Therefore, future studies should explore these relationships in other populations such as in men and in different age, BMI and ethnic groups, and different regions. Thirdly, the study participants were generally healthy (due to the exclusion criteria) and free of any chronic illness. We believe the participant characteristics may have contributed to the lack of differences in metabolic biomarkers observed between some dietary patterns. It has been discussed that measuring the concentrations of inflammatory biomarkers under basal conditions is probably less informative compared with the biomarker concentration changes in response to a challenge (e.g., nutrition intervention) [56]. Therefore, future studies should assess inflammatory responses to dietary and other physiological challenges that would provide a better indication of the impact of nutrition on inflammatory homeostasis between different population groups, including those who are at higher risk of metabolic disease. Fourthly, other confounders such as socio-economic and lifestyle factors that are known to influence diet and metabolic health were not assessed in the present study. Future studies should consider the impact of these important factors when assessing the link between diet and metabolic health. Lastly, due to the cross-sectional nature of the study, only a link between dietary patterns and metabolic health could be ascertained. Follow-up studies are needed to evaluate whether changes in certain dietary patterns lead to changes in body composition and metabolic health outcomes.

## 5. Conclusions

In this group of healthy premenopausal women, those with higher scores for the ‘refined and processed’ dietary pattern had higher BMI and adiposity, higher circulating levels of insulin and leptin, and lower levels of ghrelin compared to those with lower scores. However, longitudinal studies with multiple assessments of dietary intake, including a food-based focus, and metabolic health are required to ascertain whether this unhealthy dietary pattern may result in further adverse metabolic health outcomes over time. The findings of our study further support current recommendations to limit the consumption of refined and energy-dense foods (i.e., foods from the ‘refined and processed’ and ‘sweet and savoury snacking’ patterns), as well as encouraging the consumption of more fruit and vegetables (i.e., foods from the ‘fruit and vegetable’ pattern). Furthermore, although all women consumed food groups from all dietary patterns, we found that some dietary patterns were more strongly followed by NZE, Māori and Pacific women. The ethnic group-specific differences in dietary patterns highlight the need to investigate socio-economic and lifestyle factors that may influence the types of food chosen by different ethnic groups in order to understand specific contributors and drivers of obesity.

## Figures and Tables

**Table 1:** Characteristics of study participants (*n* = 378).

Characteristic	
Age (years)	31.0 ± 0.4
Ethnic groups *n* (%)	
NZE	225 (59%)
Māori	78 (21%)
Pacific	75 (20%)
Body weight (kg)	75.6 ± 0.9
BMI (kg/m^2^)	27.1 ± 0.3
Normal-weight BMI group (18.5–24.9 kg/m^2^) *n* (%)	
NZE	134 (59%)
Māori	27 (35%)
Pacific	11 (14%)
Overweight BMI group (25–29.9 kg/m^2^) *n* (%)	
NZE	60 (27%)
Māori	28 (36%)
Pacific	23 (31%)
Obese BMI group (≥30 kg/m^2^) *n* (%)	
NZE	31 (14%)
Māori	23 (29%)
Pacific	41 (55%)
Total body fat (%)	34.0 ± 0.4
<35% body fat group *n* (%)	
NZE	151 (67%)
Māori	38 (49%)
Pacific	28 (37%)
>35% body fat group *n* (%)	
NZE	74 (33%)
Māori	40 (51%)
Pacific	47 (63%)

NZE, New Zealand European; BMI, body mass index. Data reported as mean ± SEM or *n* (%). BMI and body fat groups were classified according to references [34,35] respectively.

**Table 2:** Dietary patterns and factor loadings of specific food groups.

Food Groups	Factor Loading
Pattern 1—Refined and processed (*9%, α* = *0.7)*	
Crumbed and deep-fried food	0.57
Fast-food	0.57
Puddings	0.54
Fruit drinks, soft drinks and other beverages	0.53
Sweetened milk products	0.52
Refined grains	0.50
Starchy vegetables	0.44
White breads	0.42
Sweetened cereals	0.34
Savoury snack foods	0.32
Water	−0.47
Nuts and seeds	−0.44
Pattern 2—Sweet and savoury snacking (*7%, α* = *0.7*)	
Cakes and biscuits	0.61
Sweet spreads	0.56
Sweet snack foods	0.55
Savoury snack foods	0.52
Margarine	0.49
Peanut butter and peanuts	0.46
Sauces	0.46
Creamy dressings	0.46
Savoury spreads	0.45
Whole grain breads	0.44
Crackers	0.42
Low-fat cheese	0.37
High-fat cheese	0.33
Pattern 3—Fruit and vegetable (*5%, α* = *0.7*)	
Dark yellow vegetables	0.65
Green vegetables	0.63
Other non-starchy vegetables	0.62
Other fruit	0.59
Starchy vegetables	0.56
Apple, banana, orange	0.53
Soy products	0.47
Legumes	0.41
Wholegrains	0.34
Yoghurt	0.32
Tomatoes	0.31
Pattern 4—Fats and meat (*4%, α* = *0.5*)	
Fats	0.54
Red meats	0.54
Creamy dressings	0.54
Processed meats	0.51
White meats	0.46
Egg and egg dishes	0.38
Coconut fats	0.37
Sauces	0.35
Green vegetables	0.35
Other alcoholic beverages	0.32
High-fat cheese	0.32
Full-fat milk	0.30
Low-fat milk	−0.42

All food groups were derived from the 220-item semi-quantitative food frequency questionnaire. Dietary patterns were extracted using the principal component analysis method and varimax rotation. Only factor loadings >0.3 are reported. Percentage variance explained by each dietary pattern and Cronbach’s α shown in brackets. *n* = 378.

**Table 3:** Age, ethnicity and body composition characteristics of dietary pattern tertiles.

	Refined and Processed Pattern			Sweet and Savoury Snacking Pattern			Fruit and Vegetable Pattern			Fats and Meat Pattern		
	T1	T2	T3	T1	T2	T3	T1	T2	T3	T1	T2	T3
Age (years)	34.4 ± 1.3	32.4 ± 1.0	27.6 ± 0.7 ^bbb,ccc^	29.6 ± 0.8	29.6 ± 0.9	32.2 ± 1.1	31.2 ± 0.8	31.0 ± 0.9	28.8 ± 0.9	29.7 ± 1.0	30.5 ± 0.9	30.4 ± 0.8
Ethnic group *n* (%)												
NZE	101 (45%)	89 (39%)	35 (16%) ^bbb,c^	54 (24%)	82 (36%)	89 (40%) ^bb^	62 (28%)	83 (37%)	80 (36%)	88 (39%)	76 (34%)	61 (27%) ^bb,c^
Māori	18 (23%)	20 (26%)	40 (51%) ^b^	42 (54%)	24 (31%)	12 (15%) ^bbb^	33 (42%)	26 (33%)	19 (24%)	16 (21%)	25 (32%)	37 (47%) ^bb^
Pacific	7 (9%)	17 (23%)	51 (68%) ^bbb,c^	30 (40%)	20 (27%)	25 (33%)	31 (41%)	17 (23%)	27 (36%)	22 (29%)	25 (33%)	28 (37%)
BMI (kg/m^2^)	25.9 ± 0.8	28.3 ± 0.6	29.9 ± 0.5 ^bbb^	28.1 ± 0.5	28.1 ± 0.6	30.1 ± 0.7	28.2 ± 0.5	28.2 ± 0.6	28.5 ± 0.6	27.1 ± 0.6	28.4 ± 0.6	29.6 ± 0.5 ^bb^
Total body fat (%)	31.9 ± 1.2	35.3 ± 0.9	37.3 ± 0.7 ^bbb^	34.0 ± 0.7	34.8 ± 0.8	37.3 ± 1.0 ^b^	34.8 ± 0.8	35.5 ± 0.9	34.5 ± 0.9	33.9 ± 0.9	34.4 ± 0.8	36.6 ± 0.7
Android fat (%)	32.0 ± 1.1	35.3 ± 0.9	36.4 ± 0.7 ^bb^	34.4 ± 0.7	34.5 ± 0.8	36.0 ± 0.9	34.6 ± 0.7	35.2 ± 0.8	34.6 ± 0.8	33.1 ± 0.9	34.9 ± 0.8	35.9 ± 0.7 ^b^
Gynoid fat (%)	36.1 ± 0.7	38.1 ± 0.6	38.2 ± 0.5	36.9 ± 0.5	37.4 ± 0.5	38.6 ± 0.6	37.8 ± 0.5	37.5 ± 0.6	37.1 ± 0.5	36.8 ± 0.6	37.5 ± 0.5	37.9 ± 0.5
WC (cm)	79.0 ± 1.7	84.7 ± 1.3 ^a^	87.6 ± 1.0 ^bbb^	83.3 ± 1.1	84.3 ± 1.2	87.8 ± 1.4 ^b^	84.1 ± 1.1	84.5 ± 1.3	84.4 ± 1.3	82.1 ± 1.3	84.1 ± 1.2	87.2 ± 1.1 ^bb^
HC (cm)	104.6 ± 1.6	109.5 ± 1.2 ^a^	111.3 ± 1.0 ^bb^	108.1 ± 1.0	107.7 ± 1.1	112.8 ± 1.3 ^b,c^	108.9 ± 1.0	108.8 ± 1.2	108.7 ± 1.1	106.8 ± 1.2	108.3 ± 1.1	111.3 ± 1.0 ^b^
WHR	0.75 ± 0.01	0.77 ± 0.01 ^a^	0.79 ± 0.01 ^b,c^	0.77 ± 0.01	0.78 ± 0.01	0.78 ± 0.01	0.77 ± 0.01	0.77 ± 0.01	0.77 ± 0.01	0.77 ± 0.01	0.77 ± 0.01	0.78 ± 0.01

T1, T2, T3, dietary pattern tertiles 1, 2 and 3; NZE, New Zealand European; BMI, body mass index; WC, waist circumference; HC, hip circumference; WHR, waist to hip circumference ratio. Data reported as estimated marginal means ± SEM or *n* (%). Differences between tertiles for continuous variables were tested by analysis of covariance (covariates—ethnic group, age) and post hoc test (with the Bonferroni correction). Differences between categorical variables were tested by chi-square test and standardised residuals. Tertile 1 vs. 2, ^a^ *p* < 0.05. Tertile 1 vs. 3, ^b^ *p* < 0.05, ^bb^ *p* < 0.01, ^bbb^ *p* < 0.001. Tertile 2 vs. 3, ^c^ *p* < 0.05, ^ccc^ *p* < 0.001. *n* = 126 in each tertile.

**Table 4:** Energy and macronutrient intakes of dietary pattern tertiles.

	Refined and Processed Pattern			Sweet and Savoury Snacking Pattern			Fruit and Vegetable Pattern			Fats and Meat Pattern		
	T1	T2	T3	T1	T2	T3	T1	T2	T3	T1	T2	T3
Total energy (MJ)	8.5 ± 0.5	8.5 ± 0.4	11.8 ± 0.3 ^bbb,ccc^	7.7 ± 0.3	10.6 ± 0.3 ^aaa^	12.7 ± 0.4 ^bbb,ccc^	8.8 ± 0.3	10.2 ± 0.4 ^a^	11.6 ± 0.4 ^bbb,c^	8.9 ± 0.4	8.7 ± 0.3	12.1 ± 0.3 ^bbb,ccc^
Protein (g)	96.5 ± 5.8	93.5 ± 4.4	121.2 ± 3.5 ^bbb,ccc^	90.7 ± 3.4	113.1 ± 4.0 ^aaa^	123.7 ± 4.5 ^bbb^	90.9 ± 3.5	108.7 ± 4.1 ^aa^	127.1 ± 4.0 ^bbb,cc^	87.7 ± 4.0	94.0 ± 3.5	133.5 ± 3.3 ^bbb,ccc^
Protein (%) (15–25%) *	19.5 ± 0.5	19.0 ± 0.4	17.5 ± 0.3 ^bbc^	20.0 ± 0.3	18.0 ± 0.4 ^aaa^	16.6 ± 0.4 ^bbb,cc^	17.8 ± 0.3	18.4 ± 0.4	19.0 ± 0.4 ^b^	16.7 ± 0.4	18.7 ± 0.3 ^aaa^	19.3 ± 0.3 ^bbb^
Fat (g)	90.1 ± 5.8	82.2 ± 4.4	108.7 ± 3.5 ^b,ccc^	72.7 ± 3.1	102.3 ± 3.6 ^aaa^	124.8 ± 4.1 ^bbb,ccc^	89.3 ± 3.6	100.3 ± 4.2	105.7 ± 4.1 ^bb^	80.0 ± 3.9	81.0 ± 3.4	124.4 ± 3.2 ^bbb,ccc^
Fat (%) (20–35%) *	38.7 ± 1.0	35.2 ± 0.7 ^a^	34.3 ± 0.6 ^bbb^	34.9 ± 0.6	35.7 ± 0.7	36.8 ± 0.8	37.3 ± 0.6	36.1 ± 0.7	33.3 ± 0.7 ^bbb,cc^	33.0 ± 0.7	34.8 ± 0.6	38.6 ± 0.6 ^bbb,ccc^
Carbohydrate (g)	181.9 ± 14.9	210.1 ± 11.2	316.1 ± 9.0 ^bbb,ccc^	190.5 ± 8.7	266.4 ± 10.2 ^aaa^	322.2 ± 11.6 ^bbb,ccc^	217.1 ± 9.6	253.3 ± 11.3	300.2 ± 11.1 ^bbb,cc^	245.8 ± 12.2	226.7 ± 10.8	283.2 ± 10.0 ^bbb^
Carbohydrate (%) (45–65%) *	36.7 ± 1.0	42.1 ± 0.8 ^aaa^	45.5 ± 0.6 ^bbb,cc^	41.5 ± 0.7	42.4 ± 0.8	43.0 ± 0.9	41.4 ± 0.7	41.7 ± 0.8	44.0 ± 0.8 ^b^	46.8 ± 0.8	42.9 ± 0.7 ^aaa^	38.3 ± 0.6 ^bbb,ccc^
Starch (%)	16.5 ± 0.9	20.2 ± 0.7 ^aa^	22.4 ± 0.5 ^bbb,c^	19.4 ± 0.5	20.6 ± 0.6	22.8 ± 0.7 ^bbb^	21.7 ± 0.6	20.0 ± 0.7	20.1 ± 0.6	23.1 ± 0.7	21.1 ± 0.6	18.6 ± 0.5 ^bbb cc^
Total sugar (%)	20.2 ± 0.9	22.0 ± 0.7	23.1 ± 0.6 ^b^	22.1 ± 0.6	21.8 ± 0.7	20.1 ± 0.8	19.7 ± 0.6	21.7 ± 0.7	23.9 ± 0.6 ^bbb^	23.7 ± 0.7	21.8 ± 0.6	19.7 ± 0.6 ^bbb,c^
Glucose (%)	4.4 ± 0.3	4.6 ± 0.2	4.6 ± 0.2	4.7 ± 0.2	4.6 ± 0.2	3.8 ± 0.2 ^bbb,c^	3.7 ± 0.2	4.5 ± 0.1 ^aa^	5.2 ± 0.2 ^bbb,c^	4.7 ± 0.2	4.4 ± 0.1	4.2 ± 0.2
Fructose (%)	4.4 ± 0.3	4.8 ± 0.2	5.1 ± 0.2	5.1 ± 0.2	4.6 ± 0.2	4.1 ± 0.3 ^b^	3.7 ± 0.2	4.7 ± 0.2 ^aa^	5.7 ± 0.2 ^bbb,cc^	5.3 ± 0.2	4.6 ± 0.2	4.2 ± 0.2 ^bb^
Sucrose (%)	7.6 ± 0.5	8.4 ± 0.4	9.6 ± 0.3 ^bb,c^	8.2 ± 0.3	8.7 ± 0.4	8.7 ± 0.4	8.4 ± 0.3	8.6 ± 0.4	8.6 ± 0.4	9.0 ± 0.4	8.7 ± 0.3	8.0 ± 0.3
Lactose (%)	3.3 ± 0.4	3.8 ± 0.3	3.4 ± 0.3	3.8 ± 0.3	3.5 ± 0.3	3.0 ± 0.3	3.3 ± 0.3	3.5 ± 0.3	4.0 ± 0.3	4.1 ± 0.3	3.6 ± 0.3	2.8 ± 0.3 ^bb^
Maltose (%)	0.43 ± 0.03	0.41 ± 0.03	0.53 ± 0.02 ^cc^	0.40 ± 0.02	0.48 ± 0.02	0.57 ± 0.03 ^bbb,c^	0.54 ± 0.02	0.46 ± 0.02	0.43 ± 0.02 ^bb^	0.55 ± 0.03	0.48 ± 0.02	0.41 ± 0.02 ^bbb,c^

Percentage energy from macronutrients calculated as a % of total energy intake. T1, T2, T3, dietary pattern tertiles 1, 2 and 3. * Acceptable macronutrient distribution range [36]. Data reported as estimated marginal means ± SEM. Differences between tertiles tested by analysis of covariance (covariates—ethnic group, age) and post hoc test (with the Bonferroni correction). Tertile 1 vs. 2, ^a^ *p* < 0.05, ^aa^ *p* < 0.01, ^aaa^ *p* < 0.001. Tertile 1 vs. 3, ^b^ *p* < 0.05, ^bb^ *p* < 0.01, ^bbb^ *p* < 0.001. Tertile 2 vs. 3, ^c^ *p* < 0.05, ^cc^ *p* < 0.01, ^ccc^ *p* < 0.001. *n* = 126 in each tertile.

**Table 5:** Metabolic and endocrine biomarkers of dietary pattern tertiles.

	Refined and Processed Pattern			Sweet and Savoury Snacking Pattern			Fruit and Vegetable Pattern			Fats and Meat Pattern		
	T1	T2	T3	T1	T2	T3	T1	T2	T3	T1	T2	T3
Leptin (ng/mL)	5.50 ± 0.001	8.51 ± 0.001 ^aa^	10.96 ± 0.001 ^bbb^	7.24 ± 0.001	9.33 ± 0.001	10.00 ± 0.001 ^b^	9.12 ± 0.001	8.91 ± 0.001	7.41 ± 0.001	7.94 ± 0.001	8.51 ± 0.001	8.91 ± 0.001
Ghrelin (pg/mL)	59.83 ± 5.93	42.56 ± 4.44	40.96 ± 3.69 ^b^	46.14 ± 3.61	49.15 ± 4.24	41.20 ± 4.82	44.21 ± 3.73	45.79 ± 4.36	47.26 ± 4.25	44.24 ± 4.54	49.49 ± 4.09	42.39 ± 3.74
Insulin (3–25 mU/mL) *	9.52 ± 1.12	12.94 ± 0.84 ^a^	16.50 ± 0.70 ^bbb,cc^	13.43 ± 0.71	14.52 ± 0.84	13.85 ± 0.95	14.78 ± 0.72	13.52 ± 0.85	13.17 ± 0.83	13.37 ± 0.88	13.88 ± 0.79	14.06 ± 0.73
Glucose (3.5–5.4 mmol/L) *	4.66 ± 0.06	4.76 ± 0.05	4.76 ± 0.04	4.74 ± 0.04	4.77 ± 0.04	4.64 ± 0.05	4.76 ± 0.04	4.72 ± 0.04	4.66 ± 0.04	4.66 ± 0.05	4.73 ± 0.04	4.80 ± 0.04
HbA1c (<40 mmol/mol) *	28.81 ± 0.52	29.05± 0.39	29.89 ± 0.32	29.44 ± 0.32	29.27 ± 0.37	29.46 ± 0.42	29.62 ± 0.32	28.91 ± 0.38	29.51 ± 0.37	29.48 ± 0.39	29.64 ± 0.35	29.22 ± 0.32
Total cholesterol (<5 mmol/L) *	4.59 ± 0.13	4.51 ± 0.10	4.45 ± 0.08	4.51 ± 0.08	4.45 ± 0.10	4.39 ± 0.11	4.40 ± 0.08	4.52 ± 0.10	4.44 ± 0.10	4.31 ± 0.10	4.54 ± 0.09	4.54 ± 0.08
HDL-C (>1 mmol/L) *	1.73 ± 0.06	1.46 ± 0.04 ^aaa^	1.41 ± 0.04 ^bbb^	1.49 ± 0.04	1.54 ± 0.04	1.46 ± 0.05	1.50 ± 0.04	1.48 ± 0.04	1.52 ± 0.04	1.51 ± 0.05	1.50 ± 0.04	1.52 ± 0.04
LDL-C (0–3.4 mmol/L) *	2.49 ± 0.13	2.57 ± 0.09	2.59 ± 0.08	2.59 ± 0.08	2.46 ± 0.09	2.48 ± 0.10	2.53 ± 0.08	2.55 ± 0.09	2.48 ± 0.09	2.38 ± 0.10	2.61 ± 0.09	2.56 ± 0.08
Triglyceride (<2 mmol/L) *	0.82 ± 0.03	1.07 ± 0.08 ^a^	1.00 ± 0.05	0.95 ± 0.05	0.98 ± 0.08	0.95 ± 0.04	0.90 ± 0.04	1.05 ± 0.08	0.94 ± 0.05	0.92 ± 0.04	0.97 ± 0.08	1.00 ± 0.05
CRP (0–5 mg/L) *	3.31 ± 1.07	3.72 ± 1.05	3.47 ± 1.05	3.39 ± 1.05	3.39 ± 1.05	3.80 ± 1.05	3.47 ± 1.05	3.63 ± 1.05	3.47 ± 1.05	3.47 ± 1.05	3.39 ± 1.05	3.55 ± 1.05
IL-6 (pg/mL)	1.95 ± 1.10	2.09 ± 1.07	2.14 ± 1.05	2.00 ± 1.05	1.95 ± 1.07	2.34 ± 1.07	2.34 ± 1.05	2.00 ± 1.07	1.86 ± 1.07	2.14 ± 1.07	1.95 ± 1.07	2.04 ± 1.07
IL-10 (pg/mL)	12.59 ± 1.17	9.55 ± 1.12	12.02 ± 1.10	10.47 ± 1.10	10.72 ± 1.12	13.49 ± 1.12	11.22 ± 1.10	12.59 ± 1.12	10.72 ± 1.12	13.49 ± 1.12	9.33 ± 1.10	10.96 ± 1.10
TNF-α (pg/mL)	6.81 ± 0.37	7.35 ± 0.28	7.36 ± 0.23	7.18 ± 0.23	7.36 ± 0.27	7.07 ± 0.31	7.41 ± 0.23	7.42 ± 0.27	6.61 ± 0.26	7.14 ± 0.29	7.11 ± 0.26	7.12 ± 0.24

T1, T2, T3, dietary pattern tertiles 1, 2 and 3; HbA1c, glycosylated haemoglobin; HDL-C, high-density lipoprotein cholesterol; LDL-C, low-density lipoprotein cholesterol; CRP, C-reactive protein; IL-6, interleukin-6; IL-10, interleukin 10; TNF-α, tumour necrosis factor-alpha. * Normal healthy ranges for metabolic biomarkers [32]. Data reported as estimated marginal means ± SEM. Differences between tertiles tested by analysis of covariance (covariates—ethnic group, age) and post hoc test (with the Bonferroni correction). Tertile 1 vs. 2, ^a^ *p* < 0.05, ^aa^ *p* < 0.01, ^aaa^ *p* < 0.001. Tertile 1 vs. 3, ^b^ *p* < 0.05, ^bbb^ *p* < 0.001. Tertile 2 vs. 3, ^cc^ *p* < 0.01. *n* = 126 in each tertile.

## Supplementary Materials

The following are available online at https://www.mdpi.com/2072-6643/11/7/1643/s1, Table S1: Food groups used in dietary pattern analysis and Table S2: Characteristics of study participants by ethnic group.
