# Supplementary material for: Dietary Patterns in New Zealand Women: Evaluating Differences in Body Composition and Metabolic Biomarkers
Source: Nutrients. 2019 Jul 18;11(7):1643. doi: 10.3390/nu11071643 (PMC6682986; doi:10.3390/nu11071643)
Supplement: Supplementary file 1 [file nutrients-11-01643-s001.zip › Supplementary table 1_Nutrients 521549.docx]

Supplementary Materials: Dietary Patterns in New Zealand Women: Evaluating Differences in Body Composition and Metabolic Biomarkers

**Shakeela N. Jayasinghe, Bernhard H. Breier, Sarah A. McNaughton, Aaron P. Russell, Paul A. Della Gatta, Shaun Mason, Welma Stonehouse, Daniel C.I. Walsh and Rozanne Kruger**

**Table S1.** Food groups used in dietary pattern analysis

| Food group | Food items |
| --- | --- |
| Full-fat milk | Full-fat milk (purple and dark blue top) |
| Low-fat milk | Lite and trim milk |
| Soy milk | Soy milk, almond milk (all non-dairy milk) |
| Sweetened milk products | Flavoured milk, fermented or evaporated milk, breakfast drinks, hot chocolate drinks, milo |
| Yoghurt | Yoghurt (plain, flavoured, Greek) |
| High-fat cheese | Cheddar, processed cheese, cream cheese, blue vein |
| Low-fat cheese | Edam, feta, mozzarella, camembert, cottage cheese |
| Apple, banana, orange | Apple, banana, orange |
| Other fruit | Al other fruit (fresh, canned, frozen, dried) |
| Tomatoes | Tomatoes |
| Dark-yellow vegetables | Pumpkin, carrot |
| Green vegetables | Green beans, silver beet, spinach, sprouts, cauliflower, brussel sprouts, cabbage, courgette, lettuce, other green leafy vegetables |
| Other non-starchy vegetables | Frozen vegetables, mushrooms, beetroot, onions, capsicum |
| Potatoes | Potato (boiled, mashed, baked, roasted) |
| Starchy vegetables | Kumara, sweet corn, taro, cassava, breadfruit, green bananas, turnips, swedes, parsnip, yams |
| White breads | Plain white bread, fruit bread, wraps, focaccia, bagel, pita, paraoa parai, rewena bread, doughboys |
| Discretionary breads | Crumpet, scone, savoury muffin, croissant, waffles, pancakes, iced buns |
| Crackers | Crackers (cream crackers, cruskits, rice crackers, vitawheat) |
| Whole grain breads | High fibre white bread, wholemeal bread, wholegrain bread |
| Refined grains | White rice, pasta, spaghetti, vermicelli, canned spaghetti, noodles (instant, egg, rice) |
| Wholegrains | Brown rice, quinoa, couscous, bulgur wheat |
| Oats | Porridge, rolled oats, oat bran, oat meal |
| Sweetened cereals | Sultana Bran, light and fruity cereal, chocolate based cereals, nutrigrain, fruit loops |
| Red meats | Beef (mince, casserole, stir-fry, roast, chop, steak, schnitzel), lamb, hogget or mutton mixed dishes (stews, casserole, stir-fry, roast, chops, steak), offal, venison |
| White meats | Pork (roast, chop, steak), Chicken (legs, wing, drumstick, breast, casserole, stir-fry), turkey, mutton bird, duck, veal |
| Processed meats | Sausages, frankfurters, saveloys, bacon, ham, luncheon meat, salami, chorizo, corned beef |
| Fish and seafood | Canned Salmon, canned tuna, canned mackerel, snapper, tarakihi, hoki, cod, gurnard, kahawai, lemon fish /shark, tuna, salmon, shrimp, crab, scallops, pipi, Kina, whitebait, roe |
| Egg and egg dishes | Whole eggs (hard-boiled, poached, fried, mashed, omelette, scrambled), mixed egg dish (quiche, frittata) |
| Legumes | Dahl, canned or dried legumes, beans (baked beans, chickpeas, lentils, peas, beans), hummus |
| Soy products | Soybeans, tofu |
| Peanut butter and peanuts | Peanut butter and peanuts |
| Nuts and seeds | Brazil nuts, walnuts, other nuts (almonds, cashew, pistachio, macadamia), seeds (pumpkin, sunflower) |
| Fats | Butter, lard, drippings |
| Coconut fats | Coconut cream and milk |
| Oil and oil based dressings | Avocado, salad dressing (Italian, French), oil (canola, olive oil) |
| Margarine | Margarine |
| Creamy dressings | Sour cream, mayonnaise, creamy dressings (aioli, tartar sauce), low-fat/calorie dressing (reduced fat mayonnaise), white sauce, cheese sauce |
| Sauces | Instant soup, sauces (tomato, BBQ, sweet chilli, mint), mustard, soy sauce, chutney, gravy (homemade, instant Gravy) |
| Sweet spreads | Jam, honey, marmalade, syrup |
| Savoury spreads | Vegemite or marmite |
| Cakes and biscuits | Cakes, loaves, sweet muffins, sweet pies, pastries, tarts, doughnuts, plain biscuits, cookies (round wine, ginger nut), fancy biscuits (chocolate, cream) |
| Puddings and other deserts | Ice cream, custard, dairy food, milk puddings (semolina, instant), other puddings or desserts (sticky date pudding, pavlova), jelly, ice blocks |
| Sweet snack foods | Lollies, chocolate, muesli bars |
| Savoury snack foods | Potato crisps, corn chips, twisties |
| Crumbed and deep-fried food | Crumbed chicken (nuggets, patties, schnitzel), frozen crumbed fish (patties, fillets, cakes, fingers, nuggets), hot potato chips, kumara chips, French fries, wedges, battered fish, fried chicken (KFC, country fried chicken) |
| Fast-food | Meat pie, sausage roll, Chinese, Indian, Thai, pizza, burgers, bread based (kebab, sandwiches, wraps, pita pit, subway) |
| Fruit and vegetable juice | Fruit juice, vegetable juice |
| Fruit drinks, soft drinks and other beverages | Fruit drink, soft drinks, iced tea, cordial, energy drinks, sports drinks, flavoured water, sparkling grape juice |
| Diet drinks | Low calorie cordial, sugar-free energy drinks, diet soft drinks |
| Tea | Tea and herbal tea |
| Coffee | Coffee instant, specialty coffees (flat white, cappuccino, lattes), decaffeinated coffee |
| Beer | Beer (standard and low alcohol) |
| Wine | Red and white wine |
| Water | Water (unflavoured mineral water, soda water, tap water) |
| Spirts and other alcoholic beverages | Sherry, spirits, liqueurs, ready-to-drink alcohol (KGB, vodka cruiser), cider, kava |
| Sugar added to food and drink | Sugar added to food and drink |

All food groups derived from the 220-item food frequency questionnaire.
