# Supplementary material for: Dietary Patterns in New Zealand Women: Evaluating Differences in Body Composition and Metabolic Biomarkers
Source: Nutrients. 2019 Jul 18;11(7):1643. doi: 10.3390/nu11071643 (PMC6682986; doi:10.3390/nu11071643)
Supplement: Supplementary file 1 [file nutrients-11-01643-s001.zip › Supplementary table 2_Nutrients 521549.docx]

Supplementary Material: Dietary Patterns in New Zealand Women: Evaluating Differences in Body Composition and Metabolic Biomarkers

**Shakeela N. Jayasinghe, Bernhard H. Breier, Sarah A. McNaughton, Aaron P. Russell, Paul A. Della Gatta, Shaun Mason, Welma Stonehouse, Daniel C.I. Walsh and Rozanne Kruger**

**Table S2.** Characteristics of study participants by ethnic group.

|  | **NZE** | **Māori** | **Pacific** |
| --- | --- | --- | --- |
| *n* | 225 | 78 | 75 |
| Age (years) | 32.0 ± 0.5 | 30.1 ± 1.0 | 29.1 ± 1.1^b^ |
| Body weight (kg) | 70.3 ± 1.0 | 77.6 ± 2.0^aa^ | 89.4 ± 2.2^bbb ccc^ |
| BMI (kg/m^2^) | 25.2 ± 0.3 | 28.0 ± 0.7^aaa^ | 31.9 ± 0.7^bbb ccc^ |
| BMI groups *n* (%)* |  |  |  |
| Normal-weight | 134 (59%) | 27 (35%) | 11 (14%) |
| Overweight | 60 (27%) | 28 (36%) | 23 (31%) |
| Obese | 31 (14%) | 23 (29%) | 41 (55%) |
| Total body fat (%) | 32.6 ± 0.5 | 34.5 ± 0.9 | 37.8 ± 0.9^bbb c^ |
| Body fat groups *n* (%) |  |  |  |
| <35% | 151 (67%) | 38 (49%) | 28 (37%) |
| >35% | 74 (33%) | 40 (51%) | 47 (63%) |
| Android fat (%) | 31.8 ± 0.5 | 34.5 ± 0.9^a^ | 37.8 ± 0.8^bbb c^ |
| Gynoid fat (%) | 37.4 ± 0.3 | 36.6 ± 0.6 | 38.4 ± 0.6 |
| WC (cm) | 78.4 ± 0.7 | 83.6 ± 1.3^aa^ | 91.2 ± 1.6^bbb ccc^ |
| HC (cm) | 104.1 ± 0.7 | 107.8 ± 1.3^a^ | 114.8 ± 1.4^bbb ccc^ |
| WHR | 0.75 ± 0.01 | 0.77 ± 0.01^aa^ | 0.79 ± 0.01^bb^ |
| Leptin (ng/mL) | 6.67 ± 0.001 | 8.28 ± 0.001 | 11.51 ± 0.001^bbb c^ |
| Ghrelin (pg/mL) | 52.62 ± 2.65 | 44.57 ± 4.51 | 36.41 ± 4.72^bb^ |
| Glucose (mmol/L) | 4.63 ± 0.03 | 4.71 ± 0.05 | 4.82 ± 0.05^bb^ |
| Insulin (mU/mL) | 9.26 ± 0.53 | 13.51 ± 0.88^aaa^ | 19.21 ± 0.92^bbb ccc^ |
| HbA1c (mmol/mol) | 27.58 ± 0.23 | 29.70 ± 0.38^aaa^ | 30.74 ± 0.40^bbb^ |
| Total cholesterol (mmol/L) | 4.77 ± 0.06 | 4.42 ± 0.10^aa^ | 4.21 ± 0.10^bbb^ |
| HDL-C (mmol/L) | 1.66 ± 0.03 | 1.47 ± 0.05^aaa^ | 1.39 ± 0.05^bbb^ |
| LDL-C (mmol/L) | 2.69 ± 0.06 | 2.46 ± 0.09 | 2.40 ± 0.10^b^ |
| Triglyceride (mmol/L) | 0.92 ± 0.05 | 1.08 ± 0.06 | 0.95 ± 0.06 |
| CRP (mg/L) | 3.57 ± 1.03 | 3.56 ± 1.05 | 3.37 ± 1.05 |
| IL-6 (pg/mL) | 1.83 ± 1.05 | 2.10 ± 1.08 | 2.25 ± 1.08 |
| IL-10 (pg/mL) | 9.82 ± 1.07 | 14.42 ± 1.12^a^ | 10.52 ± 1.12 |
| TNF-alpha (pg/mL) | 6.26 ± 0.16 | 7.65 ± 0.27^aaa^ | 7.49 ± 0.29^bbb^ |

NZE, New Zealand European; BMI, body mass index; WC, waist circumference; HC, hip circumference; WHR, waist to hip circumference ratio; HbA1c, glycosylated haemoglobin; HDL-C, high density lipoprotein cholesterol; LDL-C, low density lipoprotein cholesterol; CRP, C-reactive protein; IL-6, interleukin-6; IL-10, interleukin 10; TNF-alpha, tumour necrosis factor-alpha. * Normal-weight = 18.5–24.9 kg/m^2^, overweight = 25–29.9 kg/m^2^, obese = ≥30 kg/m^2^. All data reported as mean ± SEM or *n* (%). Differences between ethnic groups were tested by one-way analysis of variance and post hoc test (with the Bonferroni correction). NZE *vs.* Māori, ^a^ *p* < 0.05, ^aa^ *p* < 0.01, ^aaa^ *p* < 0.001. NZE *vs.* Pacific, ^b^ *p* < 0.05, ^bb^ *p* < 0.01, ^bbb^ *p* < 0.001. Māori *vs.* Pacific, ^c^ *p* < 0.05, ^cc^ *p* < 0.01, ^ccc^ *p* < 0.001
